# Repeated Administration of Clinical Doses of Tramadol and Tapentadol Causes Hepato- and Nephrotoxic Effects in Wistar Rats

**DOI:** 10.3390/ph13070149

**Published:** 2020-07-10

**Authors:** Joana Barbosa, Juliana Faria, Fernanda Garcez, Sandra Leal, Luís Pedro Afonso, Ana Vanessa Nascimento, Roxana Moreira, Odília Queirós, Félix Carvalho, Ricardo Jorge Dinis-Oliveira

**Affiliations:** 1 IINFACTS—Institute of Research and Advanced Training in Health Sciences and Technologies, Department of Sciences, University Institute of Health Sciences (IUCS), CESPU, CRL, 4585-116 Gandra, Portugal; juliana.faria@iucs.cespu.pt (J.F.); fernanda.garcez@cespu.pt (F.G.); sandra.leal@iucs.cespu.pt (S.L.); anavanessa65@gmail.com (A.V.N.); roxanamoreira@moreno.pt (R.M.); odilia.queiros@iucs.cespu.pt (O.Q.); 2 UCIBIO, REQUIMTE—Laboratory of Toxicology, Department of Biological Sciences, Faculty of Pharmacy, University of Porto, 4050-313 Porto, Portugal; felixdc@ff.up.pt; 3 Department of Public Health and Forensic Sciences, and Medical Education, Faculty of Medicine, University of Porto, 4200-319 Porto, Portugal; 4 Department of Biomedicine, Unit of Anatomy, Faculty of Medicine, University of Porto, 4200-319 Porto, Portugal; 5 CINTESIS—Center for Health Technology and Services Research, Faculty of Medicine, University of Porto, 4200-450 Porto, Portugal; 6 Department of Pathology, Portuguese Institute of Oncology of Porto, 4200-072 Porto, Portugal; lpafonso@gmail.com

**Keywords:** tramadol, tapentadol, prescription opioids, hepatotoxicity, nephrotoxicity, in vivo studies

## Abstract

Tramadol and tapentadol are fully synthetic and extensively used analgesic opioids, presenting enhanced therapeutic and safety profiles as compared with their peers. However, reports of adverse reactions, intoxications and fatalities have been increasing. Information regarding the molecular, biochemical, and histological alterations underlying their toxicological potential is missing, particularly for tapentadol, owing to its more recent market authorization. Considering the paramount importance of liver and kidney for the metabolism and excretion of both opioids, these organs are especially susceptible to toxicological damage. In the present study, we aimed to characterize the putative hepatic and renal deleterious effects of repeated exposure to therapeutic doses of tramadol and tapentadol, using an in vivo animal model. Male Wistar rats were randomly divided into six experimental groups, composed of six animals each, which received daily single intraperitoneal injections of 10, 25 or 50 mg/kg tramadol or tapentadol (a low, standard analgesic dose, an intermediate dose and the maximum recommended daily dose, respectively). An additional control group was injected with normal saline. Following 14 consecutive days of administration, serum, urine and liver and kidney tissue samples were processed for biochemical, metabolic and histological analysis. Repeated administration of therapeutic doses of both opioids led to: (i) increased lipid and protein oxidation in liver and kidney, as well as to decreased total liver antioxidant capacity; (ii) decreased serum albumin, urea, butyrylcholinesterase and complement C3 and C4 levels, denoting liver synthesis impairment; (iii) elevated serum activity of liver enzymes, such as alanine aminotransferase, aspartate aminotransferase, alkaline phosphatase and γ-glutamyl transpeptidase, as well as lipid profile alterations, also reflecting hepatobiliary commitment; (iv) derangement of iron metabolism, as shown through increases in serum iron, ferritin, haptoglobin and heme oxygenase-1 levels. In turn, elevated serum cystatin C, decreased urine creatinine output and increased urine microalbumin levels were detected upon exposure to tapentadol only, while increased serum amylase and urine *N*-acetyl-β-D-glucosaminidase activities were observed for both opioids. Collectively, these results are compatible with kidney injury. Changes were also found in the expression levels of liver- and kidney-specific toxicity biomarker genes, upon exposure to tramadol and tapentadol, correlating well with alterations in lipid profile, iron metabolism and glomerular and tubular function. Histopathological analysis evidenced sinusoidal dilatation, microsteatosis, mononuclear cell infiltrates, glomerular and tubular disorganization, and increased Bowman’s spaces. Although some findings are more pronounced upon tapentadol exposure, our study shows that, when compared with acute exposure, prolonged administration of both opioids smooths the differences between their toxicological effects, and that these occur at lower doses within the therapeutic range.

## 1. Introduction

Opioid drugs that produce morphine-like effects by interacting with opioid receptors are a cornerstone of moderate to severe, malignant and non-malignant pain treatment, both in acute and chronic settings [1,2,3,4,5]. Their widespread prescription, abuse, misuse and related mortality has increased in developed countries, contributing to an “opioid crisis” in the United States of America and reinforcing the scrutiny over their benefit-risk balance [6,7,8,9,10,11]. In other continents, including Asia, Northern and Western Europe, albeit the situation is not as dramatic, it is still raising serious public health issues and awareness [3,4,5,6,7,9,12,13,14,15,16,17,18].

Tramadol ((1*RS*,2*RS*)-2-[(dimethylamino)methyl]-1-(3-methoxyphenyl)-cyclohexanol) and tapentadol (3-[(1*R*,2*R*)-3-(dimethylamino)-1-ethyl-2-methylpropyl]phenol) are fully synthetic analgesic opioids that synergistically combine mu-opioid receptor (MOR) agonism with monoamine reuptake inhibition, justifying their classification as “atypical opioids” [1,2,11,19,20,21,22,23,24,25,26]. Such dual mechanism of action optimizes analgesia and minimizes opioid-typical side effects, such as drowsiness, nausea, vomiting, constipation, motor incoordination and respiratory depression [1,2,11,19,27], explaining their indications for the treatment of post-surgical, musculoskeletal, inflammatory, cancer and neuropathic pain, as well as mixed pain states [1,19,27,28,29,30,31,32]. Also, owing to the synergistic combination of their mechanisms of action, these opioids allow the dose administered to be reduced without compromising analgesic efficacy, thus reducing the potential for abuse and addiction [11,33].

Tramadol is commercially available as a racemate; while (+)-tramadol provides for serotonin (5-HT) reuptake inhibition, (−)-tramadol accounts for noradrenaline (NA) uptake inhibition [1,2,11,19,32,34,35,36,37]. It undergoes extensive hepatic metabolism, mainly through O- and N-demethylation and conjugation reactions, yielding at least 14 phase I and 12 phase II metabolites [1,2,11,35,37,38,39]. Ninety percent of racemic tramadol elimination is ensured by the kidneys, with an elimination half-life of 5–6 h [1,2,19,35,38].

Tapentadol has been developed from the structures of tramadol, *O*-desmethyltramadol and morphine, having been more recently made available on the market [1,40,41]. It acts mainly on NA reuptake inhibition and has minimal 5-HT activity, thus minimizing serotonin syndrome liability [1,2,25,27,30,32,33,36,39,42,43,44,45]. It is metabolized mainly through phase II glucuronidation and sulphonation reactions [1,2,25,36,39,43,44,45]. Kidneys are also the major elimination route for tapentadol, accounting for 99% of its excretion; its elimination half-life is about 4 or 5–6 h (for immediate and prolonged release formulations, respectively) [1,2,38,39,44,46]. Its mu-load, i.e., the contribution of the opioid component to the adverse effect magnitude, in relation to pure MOR agonists at equianalgesia, has been estimated as ≤40% [30,43]. It is argued to be an upgrade of comparable opioids, particularly tramadol, whose drawbacks have inspired tapentadol design [1,2]. However, its shorter market history limits the amount of clinical and toxicological information on its use, hindering a true comparison between both opioids [1,2,32,42].

Although tramadol and tapentadol are claimed to have better safety profiles than their opioid peers, several adverse events have been reported, including nausea, vomiting, dizziness, seizures, dyspnea, respiratory depression [1,2,14,47,48,49,50,51], and even fatal cases [10,42,51,52,53,54,55,56,57,58,59,60,61,62,63,64,65,66,67,68]. In the VigiBase™ World Health Organization (WHO) Global Database of Individual Case Safety Reports concerning 5-HT toxicity, tramadol ranks 1st and tapentadol ranks 3rd (with 647 and 115 cases out of 1641, respectively) as the only suspected cause or amongst other drugs, and 1st and 2nd (with 62 and 42 cases out of 147, respectively) as the only suspected cause [34,69]. In addition, in spite of their theoretically lower potential for abuse and dependence, cases of misuse, dependence and addiction have been reported [1,2,10,11,14,60,70]. Therefore, while their public health burden is reported to be low, it is not absent [10,33].

Considering the roles of liver and kidney on tramadol and tapentadol metabolism and excretion, these organs are particularly liable toxicity targets. A case cross-over study addressing the period of 2004–2013 identifies an association between tramadol use and increased mortality risk, with renal and hepatic disease representing prominent risk factors [51]. Accordingly, in vivo studies document the hepato- and nephrotoxicity of various opioids, particularly tramadol, morphine, and heroin. Such studies, mainly performed in rodents, encompass several routes of administration (e.g., oral, intraperitoneal (i.p.), intramuscular, subcutaneous), exposure periods ranging from acute to chronic, and doses ranging from therapeutic ones to overdoses. All have shown liver and kidney commitment, evidenced through increased liver enzyme activities, blood urea nitrogen (BUN), creatinine [71,72,73,74,75,76,77,78,79,80,81,82,83,84,85,86,87], tissue oxidative markers (e.g., increased liver and kidney malondialdehyde (MDA) levels) [75,76,80,82,84,88,89,90], as well as through decreased antioxidant activity (e.g., decreased catalase, superoxide dismutase and glutathione peroxidase activities, decreased glutathione levels) [76,80,82,83,84,88,89,90,91]. Hepato- and nephrotoxicity were also observed at the histological level. Liver histological findings include centrilobular congestion, cytolysis and sinusoidal dilatation [71,73,74,76,77,78,79,81,84,85,87,88,92,93,94,95,96], while kidney histopathology comprises endothelial cell swelling, atrophied glomeruli with collapsed tufts, wide Bowman’s spaces and interstitial nephritis; in turn, inflammatory cell infiltration, vacuolization, degeneration, focal necrosis, hemorrhage and fibrosis have been reported for both organs [71,74,76,77,78,81,84,85,88,95].

In line with this, previous studies by our group have shown toxicological damage, using in vitro and in vivo approaches, following an acute exposure to tramadol and tapentadol [97,98,99]. In particular, hepato- and nephrotoxicity were found upon Wistar rat exposure to therapeutic doses [98]. Nevertheless, to our knowledge, no similar comparative studies concerning short-term, repeated therapeutic dose administrations, are available. In this context, in the present study, we aimed to characterize the putative hepato- and nephrotoxic effects resulting from the repeated administration of clinical doses of tramadol and tapentadol at the molecular, biochemical, and histological levels, at a subacute time point that precedes most of those assayed in comparable studies. Also, we aimed to ascertain whether these effects are intensified along with the exposure, as compared to our acute context results. The importance of this work is further underlined by opioid use on a frequently subacute, sub-chronic and chronic basis, as well as by the gap of toxicological information on tapentadol.

## 2. Results

### 2.1. Repeated Exposure to Tramadol and Tapentadol Causes Oxidative Stress and Differentially Changes the Antioxidant Status of Liver and Kidney

**Figure 1:**
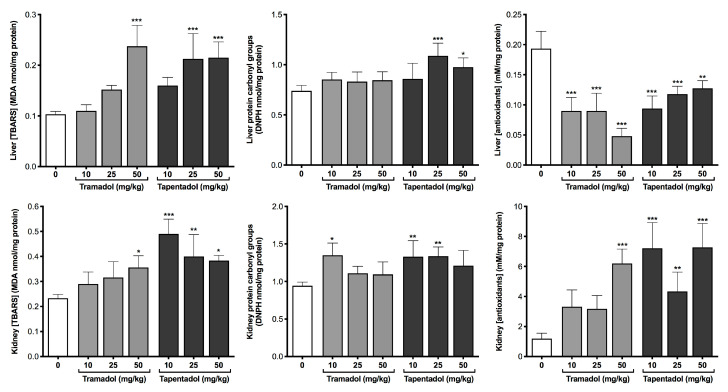
Liver and kidney oxidative stress analysis, assayed as thiobarbituric acid reactive substances (TBARS), protein carbonyl groups and total antioxidant capacity (Trolox equivalents), in Wistar rat tissue homogenates prepared upon daily intraperitoneal (i.p.) administration of 10, 25 or 50 mg/kg tramadol or tapentadol, for 14 consecutive days. Results were normalized against total protein content and are expressed by means ± SD. *** *p* < 0.001, ** *p* < 0.01, * *p* < 0.05. MDA: malondialdehyde; DNPH: 2,4-dinitrophenylhydrazine.

To characterize the effect of tramadol and tapentadol repeated administration of therapeutic doses in liver and kidney oxidative stress, thiobarbituric acid reactive substances (TBARS) and carbonyl groups, biomarkers of lipid and protein oxidative stress, respectively, were quantified in tissue homogenates. Additionally, the total antioxidant capacity was determined in the same samples, through spectrophotometry. Results are depicted in Figure 1.

Both opioids led to increased TBARS levels; while tramadol caused a significant increase at 50 mg/kg only, in both liver and kidney, tapentadol led to the same effect at 25 and 50 mg/kg in liver, and at all doses in kidney. Protein carbonyl groups increased at the intermediate and highest tapentadol doses only (liver), while in kidney such increase was observed at the lowest tramadol and lowest and intermediate tapentadol doses. In turn, the total antioxidant capacity is significantly lower in liver at all doses of both opioids, but augmented at the highest tramadol dose and at all tapentadol doses in kidney. Thus, it might be hypothesized that liver and kidney respond differently to oxidative insult and that it has a differential impact on the antioxidant status of these organs.

### 2.2. Repeated Exposure to Tramadol and Tapentadol Compromises Liver and Kidney Metabolic and Excretion Functions

**Figure 2:**
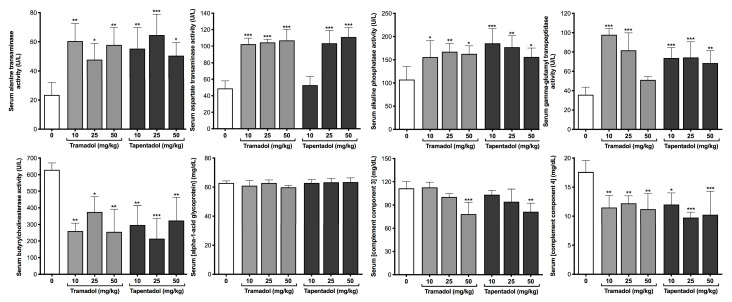
Concentrations of serum biochemical parameters, concerning liver synthetic function and liver function tests, upon Wistar rat repeated daily intraperitoneal (i.p.) administration of 10, 25 or 50 mg/kg tramadol or tapentadol, for 14 consecutive days. Results are expressed as means ± SD. *** *p* < 0.001, ** *p* < 0.01, * *p* < 0.05.

**Figure 3:**
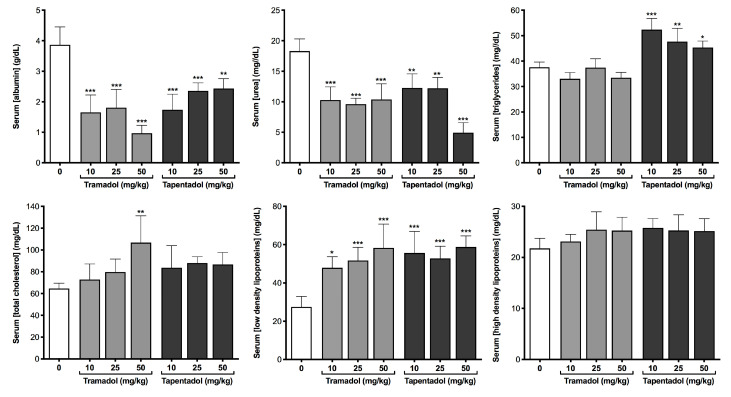
Concentrations of serum biochemical parameters, concerning liver synthetic function and lipid profile, upon Wistar rat repeated daily intraperitoneal (i.p.) administration of 10, 25 or 50 mg/kg tramadol or tapentadol, for 14 consecutive days. Results are expressed as means ± SD. *** *p* < 0.001, ** *p* < 0.01, * *p* < 0.05.

**Figure 4:**
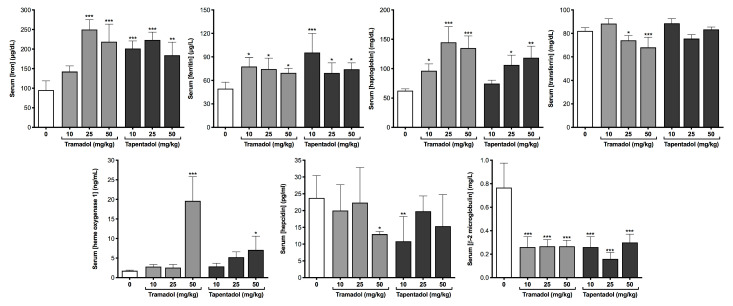
Concentrations of serum biochemical parameters, concerning iron metabolism, upon Wistar rat repeated daily intraperitoneal (i.p.) administration of 10, 25 or 50 mg/kg tramadol or tapentadol, for 14 consecutive days. Results are expressed as means ± SD. *** *p* < 0.001, ** *p* < 0.01, * *p* < 0.05.

**Figure 5:**
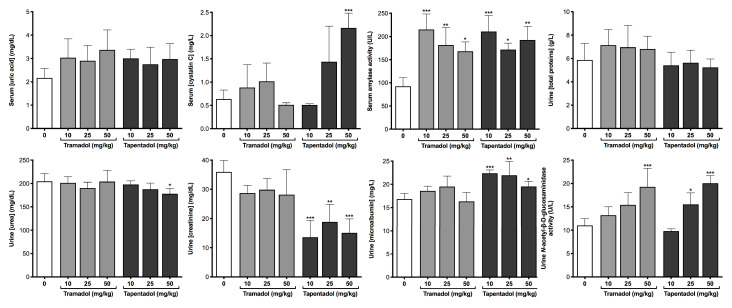
Concentrations of serum and urine biochemical parameters, concerning kidney function, upon Wistar rat repeated daily intraperitoneal (i.p.) administration of 10, 25 or 50 mg/kg tramadol or tapentadol, for 14 consecutive days. Results are expressed as means ± SD. *** *p* < 0.001, ** *p* < 0.01, * *p* < 0.05.

A battery of biochemical and immunological parameters was quantified in serum and urine samples to get an insight into the putative metabolic and inflammatory effects of the repeated exposure to tramadol and tapentadol clinical doses. Serum results are represented in Figure 2, Figure 3, Figure 4 and Figure 5, while urinary determinations appear in Figure 5 only.

Figure 2 concerns liver enzymes—alanine aminotransferase (ALT), aspartate aminotransferase (AST), alkaline phosphatase (ALP), γ-glutamyl transpeptidase (GGT) and butyrylcholinesterase (BuChE)—and immunological parameters of hepatic origin—α-1-acid glycoprotein and complement component 3 (C3) and 4 (C4) proteins. The activity of all liver enzymes, except for BuChE, was found to be significantly increased at almost all doses of both tramadol and tapentadol, with ALT activity rising around 3-fold, AST 2-fold, and ALP 1.6-fold, on average, above the control. GGT activity increased roughly 2.5-fold at 10 and 25 mg/kg tramadol, while tapentadol led to an approximate average increase of 2.0-fold, irrespectively of the dose. Although α-1-acid glycoprotein concentrations did not change in a statistically significant manner, BuChE activity and complement C4 levels decreased to about 46% and 63% of the control values, respectively, at all opioid doses. Complement C3 levels decreased significantly to about 72% of the control at the highest tramadol and tapentadol dose.

Figure 3 data illustrates liver synthetic function and lipid profile. Although serum total proteins had no statistically significant changes (results not shown), serum albumin and urea levels are markedly decreased in all experimental groups; while albumin concentration decreases to about 25% and 63% of the control values at 50 mg/kg tramadol and tapentadol, respectively, urea decreases to about 60% at all opioid doses except at 50 mg/kg tapentadol, where it reaches 27% of the control values. In turn, serum lipid parameters also denote alterations in the lipid profile. While only tapentadol leads to significant increases in triglyceride levels, total cholesterol increased solely at the highest tramadol dose. Low-density lipoprotein (LDL) cholesterol increased upon repeated administration of all opioid doses, whilst no statistically significant differences were found between control and experimental groups for high-density lipoprotein (HDL) cholesterol. Together with those from Figure 2, these data support liver damage at different levels.

Collectively, the parameters in Figure 4 reflect changes in iron metabolism. The average 2.3-fold increase in serum iron concentrations, observed at almost all opioid doses, was accompanied by increases in ferritin (1.6-fold, on average), in haptoglobin (to a maximum of 2.3-fold), and heme oxygenase 1 (HO-1, whose activity increased 11.2-fold and 4.0-fold upon exposure to 50 mg/kg tramadol and tapentadol, respectively). In addition, serum transferrin levels were found to decrease at 25 and 50 mg/kg tramadol doses, while serum hepcidin concentration significantly decreased at tramadol and tapentadol highest and lowest dose, respectively. β-2-Microglobulin (B2M) markedly decreased to about 33% of the control values, irrespectively of the opioid and dose considered.

In turn, Figure 5 shows the results concerning kidney function biomarkers. While there were no statistically significant increases in serum uric acid concentrations, serum cystatin C levels were significantly elevated at 50 mg/kg tapentadol, and amylase activity nearly doubled at all doses of both opioids. Figure 5 also encompasses data obtained from the analysis of Wistar rat urine samples. While there were no statistically significant changes in total protein concentration between control and experimental groups, urea levels significantly decreased at 50 mg/kg tapentadol, paralleling the more pronounced decrease found in serum samples from this group (Figure 3). All tapentadol doses led to a decrease in creatinine urinary elimination (with the values reaching 44% of the control) and an increase in urine microalbumin levels. In turn, tramadol highest dose and tapentadol intermediate and highest doses caused an increase in *N*-acetyl-β-D-glucosaminidase (NAG) activity (1.8-fold average increase at 50 mg/kg opioid).

Taken as a whole, Figure 5 substantiates that there are renal changes following repeated administration of tramadol and tapentadol therapeutic doses.

### 2.3. Repeated Exposure to Tramadol and Tapentadol Leads to Changes in the Gene Expression of Liver and Kidney Toxicity Biomarkers

**Figure 6:**
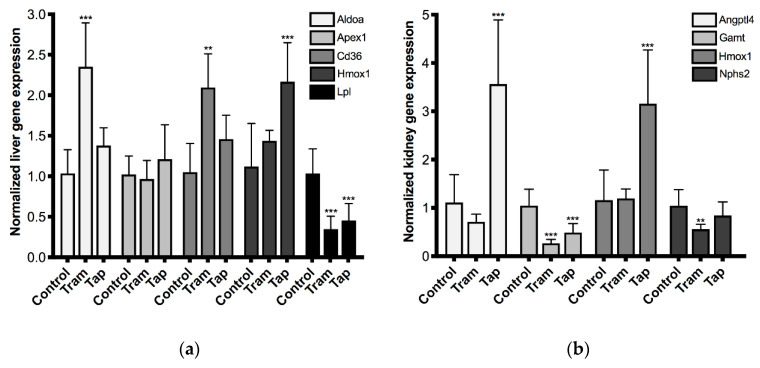
Normalized gene expression levels of liver (**a**) and kidney (**b**) toxicity biomarkers, upon Wistar rat repeated daily intraperitoneal (i.p.) administration of 50 mg/kg tramadol (Tram) or tapentadol (Tap), for 14 consecutive days. Expression levels were normalized against the respective 18S ribosomal RNA (18S rRNA) gene expression, and then against the respective controls (administered with normal saline), set as 1. Results are expressed as means ± SD. *** *p* < 0.001, ** *p* < 0.01. Aldoa: fructose-bisphosphate aldolase A; Angptl4: angiopoietin-like 4; Apex1: apurinic/apyrimidinic endonuclease 1; Cd36: cluster of differentiation 36/fatty acid translocase; Gamt: guanidinoacetate *N*-methyltransferase; Hmox1: heme oxygenase 1; Lpl: lipoprotein lipase; Nphs2: podocin.

Aiming at the characterization of the putative hepato-renal impact of the repeated exposure to tramadol and tapentadol clinical doses, a small-scale gene expression profiling was performed through quantitative Real-Time Polymerase Chain Reaction (qRT-PCR), for a selection of toxicity biomarkers (Figure 6). RNA was isolated from liver and kidney specimens from Wistar rats exposed to 50 mg/kg tramadol and tapentadol, and gene expression levels were compared to those of the control (non-treated) group.

Regarding the liver toxicity biomarker panel (Figure 6a), tramadol led to increases in fructose-bisphosphate aldolase A (Aldoa, 2.3-fold) and cluster of differentiation 36/fatty acid translocase (Cd36, 2.0-fold) gene expression, while tapentadol also approximately doubled that of heme oxygenase 1 (Hmox1). Lipoprotein lipase (Lpl) gene expression was found to be reduced upon exposure to both opioids (reaching 34% and 44% of the control for tramadol and tapentadol, respectively), whilst no significant changes were detected in apurinic/apyrimidinic endonuclease 1 (Apex1) expression. As far as the kidney panel is concerned (Figure 6b), angiopoietin-like 4 (Angptl4) and Hmox1 expression roughly triplicated upon tapentadol exposure. In turn, guanidinoacetate *N*-methyltransferase (Gamt) gene expression decreased after exposure to tramadol and tapentadol (achieving 26% and 47% of the control, respectively), while only tramadol led to a significant decrease in podocin (Nphs2) expression (reaching 53% of the control). Therefore, the expression of almost all genes under study was found to be altered upon exposure to at least one of the opioids, showing that tramadol- and tapentadol-induced hepato- and nephrotoxicity also have gene expression implications.

### 2.4. Repeated Exposure to Tramadol and Tapentadol Leads to Glycogen Depletion, Microsteatosis and Inflammation in Liver and Kidney, and to Fibrous Tissue Deposition between Hepatocytes

**Figure 7:**
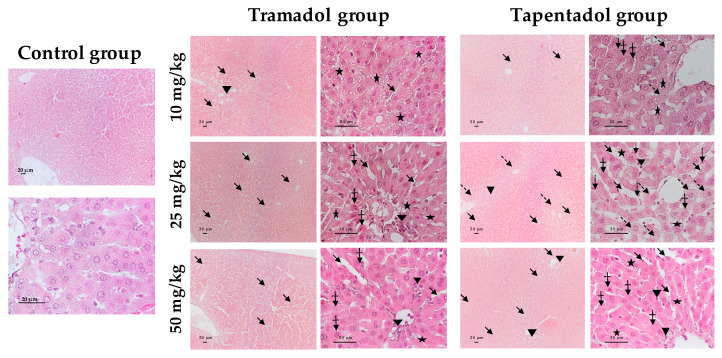
Liver sections of Wistar rats intraperitoneally injected with different tramadol and tapentadol doses or saline (control group) for 14 consecutive days, upon hematoxylin & eosin (H&E) staining. Mononuclear inflammatory infiltrates (inverted triangles), sinusoidal dilatation (arrows), vacuolization/microsteatosis (stars), fragmented nuclei/loss of definition of nuclear membranes (vertical, crossed arrows), hypopigmented areas (dashed arrows) and vascular congestion/erythrocyte extravasation (vertical, dotted arrows) are observed. Photographs were taken with 100× and 600× magnifications. Scale bar, 20 µm.

**Figure 8:**
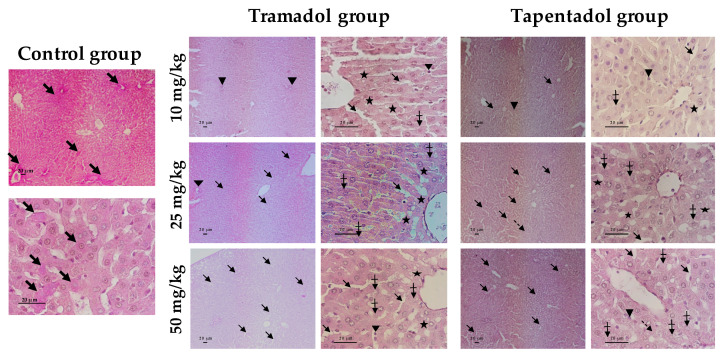
Liver sections of Wistar rats intraperitoneally injected with different tramadol and tapentadol doses or saline (control group) for 14 consecutive days, upon periodic acid-Schiff (PAS) staining. Glycogen granules appear as purple areas (thick arrows). Mononuclear inflammatory infiltrates (inverted triangles), sinusoidal dilatation (arrows), vacuolization/microsteatosis (stars), fragmented nuclei/loss of definition of nuclear membranes (vertical, crossed arrows) and hypopigmented areas (dashed arrows) are observed. Photographs were taken with 100× and 600× magnifications. Scale bar, 20 µm.

**Figure 9:**
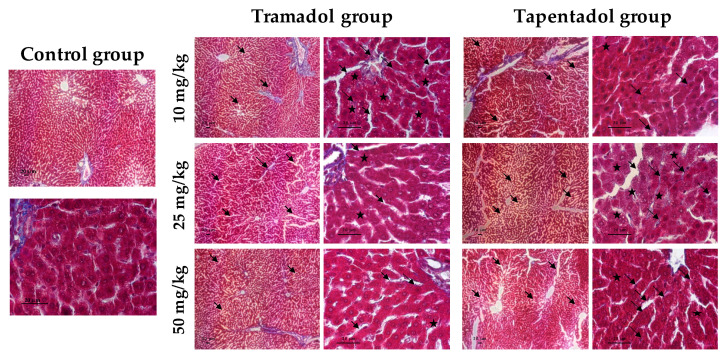
Liver sections of Wistar rats intraperitoneally injected with different tramadol and tapentadol doses or saline (control group) for 14 consecutive days, upon Masson’s trichrome staining. Sinusoidal dilatation (arrows) and vacuolization (stars) are observed. Traces of fibrous tissue (dotted arrows) are found between hepatocytes. Photographs were taken with 100× and 600× magnifications. Scale bar, 20 µm.

**Figure 10:**
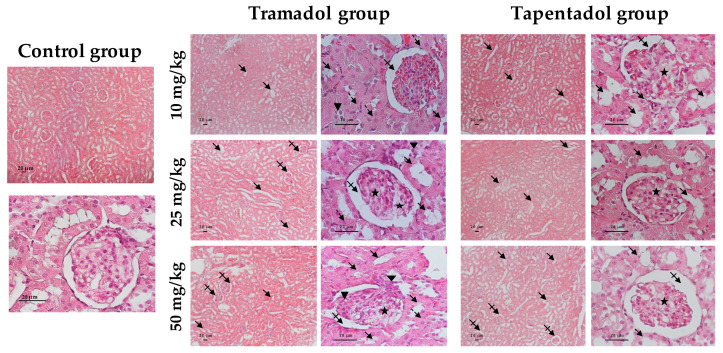
Kidney sections of Wistar rats intraperitoneally injected with different tramadol and tapentadol doses or saline (control group) for 14 consecutive days, upon hematoxylin & eosin (H&E) staining. Inflammatory mononuclear cell infiltrates (inverted triangles), increased Bowman’s spaces (crossed arrows), disorganized and vacuolized glomeruli (stars), swollen cells (dashed arrows), and disorganized and poorly contoured tubules (arrows) are observed. Photographs were taken with 100× and 600× magnifications. Scale bar, 20 µm.

The in vivo effects of repeated tramadol and tapentadol administration were also studied at the histopathological level, by comparing liver and kidney specimens from Wistar rats exposed to 10, 25 and 50 mg/kg tramadol or tapentadol with those from controls, injected with saline. Liver tissue samples were stained with hematoxylin & eosin (H&E, Figure 7), periodic acid-Schiff (PAS, Figure 8) and Masson’s trichrome (Figure 9) procedures. Kidney tissue samples were stained with H&E (Figure 10). H&E staining evidences cell nuclei as blue, extracellular matrix and cytoplasm as pink and other cell structures as different shades and combinations of these colors, providing an overview of the tissue’s structure. PAS staining, in turn, detects polysaccharides and mucosubstances, while Masson’s trichrome is a three-color protocol that stains nuclei dark red/purple, cytoplasm red/pink and connective tissue blue. 

The controls showed the typical liver tissue architecture, with polyhedral hepatocytes arranged in cords, separated by sinusoids and radiating from the central vein to the portal areas, with a granular eosinophilic cytoplasm [100]. However, all three staining methods evidenced the presence of histological alterations in liver sections from the experimental groups, such as sinusoidal dilatation and vacuolization, which was valued as microsteatosis (Figure 7, Figure 8 and Figure 9). Sinusoidal dilatation became increasingly patent along with tramadol dose, whilst it was found beyond perivascular regions, denoting more extensive damage, on tapentadol slides (Figure 7 and Figure 8). 

Mononuclear cell infiltrates were observed at all tramadol doses, although for tapentadol they became more evident along with the dose (Figure 7 and Figure 8). Some cells displayed poorly contoured nuclei, often with a fragmented appearance, upon exposure to both opioids (Figure 7 and Figure 8). Signs of vascular congestion/erythrocyte extravasation were apparent in H&E sections from the tapentadol group (Figure 7), which also produced hypopigmented areas through H&E and PAS methods (Figure 7 and Figure 8). 

All tramadol and tapentadol doses led to glycogen depletion, as inferred from the weaker purple staining in the experimental groups, when compared with the controls (Figure 8). 

In turn, Masson’s trichrome staining allowed the identification of fibrous tissue between hepatocytes at all doses of both opioids, though more abundant and thicker for tapentadol, whose dose increments seemingly intensified this effect (Figure 9).

As far as kidney sections are concerned (Figure 10), the control shows the expected histology, with glomeruli composed of capillary tufts lying within the Bowman’s capsule, from which they are separated by narrow Bowman’s spaces, and a network of proximal and distal tubules. The microscopic analysis of experimental group slides reveals that both opioids led to tubule disorganization at all doses studied. Glomeruli also appeared disorganized and vacuolated at all tapentadol doses, although for tramadol such observation became more obvious at 25 and 50 mg/kg; in contrast, increased Bowman’s spaces were seen at all tramadol doses, but were more evident at 50 mg/kg tapentadol only. In addition, tramadol exposure was associated with the presence of inflammatory cell infiltrates and swollen cells.

Thus, a combined analysis of the results from the three staining methods shows the presence of histological signs compatible with toxicological damage at all doses of both opioids. Whether these signs are dose-dependent or -independent, it varies according to the opioid and finding considered.

## 3. Discussion

Tramadol and tapentadol are two prescription opioids widely used in the treatment of moderate to severe forms of pain. Their generalized prescription is greatly due to their therapeutic efficiency and safety, owing to their synergistic and atypical mechanism of action. Nevertheless, adverse events and fatalities have been reported and, given their common use on a repeated and chronic basis, concerns about dependence liability and abuse potential have been rising. Considering that liver and kidney are central players in tramadol and tapentadol pharmacokinetics, we aimed to study their putative hepato- and nephrotoxic effects, in an in vivo model submitted to repeated administration of therapeutic doses. This is, to our knowledge, the first study addressing tramadol and tapentadol comparative toxicity upon repeated administration. The effects of an acute exposure of Wistar rats to the same doses were already reported by our own research team [98,99]. Since hepato- and nephrotoxicity have already been demonstrated on such acute settings [98], and considering that tramadol and tapentadol are often consumed for longer periods, the present study not only broadens the picture provided by our acute exposure assays, but also more closely reflects the real consumption scenario for both opioids. 

It should be stressed that, in spite of their chemical resemblance, there are differences between tramadol and tapentadol regarding receptor and transporter affinity, as well as their pharmacokinetics, metabolite profiles and pharmacodynamics, which may also account, to some extent, for different results [1,2,98,99]. The route of tramadol and tapentadol administration used in our study also deserves an additional important remark. Despite bypassing the intestine, i.p. injection resembles oral administration from a pharmacokinetic point of view, since drugs are absorbed into the mesenteric vessels draining into the portal vein [101]. Therefore, they may undergo hepatic metabolism before reaching systemic circulation. In this sense, given that the two opioids have different bioavailabilities (68–84% and 32% for tramadol and tapentadol, respectively, upon oral administration [1,2,35]), the doses used in our study, although mathematically equal, were not pharmacologically equivalent. From this perspective, to ensure pharmacological equivalence, tapentadol doses should be increased. Such approach would further accentuate differences in the results obtained for some of the parameters discussed below.

### 3.1. Repeated Exposure to Tramadol and Tapentadol Induces Hepato-Renal Oxidative Stress, Affecting Liver and Kidney Cell Integrity and Function

The association between opioid exposure and oxidative stress is well documented. Multiple studies report increased MDA levels in liver, kidney and serum upon opioid repeated administration, such as those from Awadalla, El-Gaafarawi, Elkhateeb, Ibrahim and their respective colleagues, who orally administered rats with 30 to 150 mg/kg tramadol, for 20 to 30 days [75,76,84,88,89], as well as similar studies with morphine [80,82] and heroin [90]. These studies have also associated tramadol exposure with decreased levels of antioxidant defenses, such as reduced glutathione, glutathione peroxidase, superoxide dismutase and catalase in liver and kidney tissues [76,84,88], as well as in serum [89]. Studies concerning repeated administration of morphine in mice have also led to similar results in liver [82,91]. 

In the present study, an increase in TBARS and protein carbonyl groups was found in liver and kidney homogenates, following repeated exposure to clinical doses of both opioids, particularly for tapentadol (Figure 1). While the same trend was found for protein carbonyl groups in our previous acute exposure assays [98], TBARS results were different, as their liver and kidney levels were decreased upon acute exposure [98], but increased upon repeated exposure. This suggests that the protective effect against lipid peroxidation (LPO), hypothesized for acute exposure settings [98], is lost upon repeated administration. Also, TBARS and protein carbonyl groups data may be paralleled with total antioxidant capacity results (Figure 1). It might be argued that, while hepatocytes experience increased oxidative stress as a result of a decreased antioxidant capacity (as seen through increased TBARS and protein carbonyl groups), kidney cells increase their antioxidant capacity as a response to opioid-induced oxidative protein damage, thus possibly explaining carbonyl group results, whose increase is not statistically significant for the highest doses (Figure 1). Taken together, our results show that, as for similar studies, the induction of lipid and protein oxidative stress is a toxicity mechanism associated with in vivo repeated administration of tramadol and tapentadol, even at therapeutic doses.

### 3.2. Repeated Exposure to Tramadol and Tapentadol Causes Cumulative Hepatocellular and Hepatobiliary Damage

In an attempt to further characterize the hepatic effects of tramadol exposure, several studies have also reported increased serum ALT, AST, ALP and GGT activities following repeated administration of rodents with doses ranging from 3 to 200 mg/kg, through different routes [71,72,73,74,75,76,77,78,79,81,84]. Analogous assays with morphine have led to similar results [80,82,87]. In accordance, ALT, AST, ALP and lactate dehydrogenase activities were found to be elevated among tramadol abusers [102]. In a case report concerning fatal hepatic failure following accidental tramadol overdose, ALT and AST activities increased by more than 30-fold in relation to the reference range, while GGT was close to the upper reference limit [59]. In line with this, we found serum ALT and AST activities to be increased at almost all doses of both tramadol and tapentadol (Figure 2), with the increase in ALT, a more sensitive and hepatospecific enzyme than AST [103,104,105], being higher. Such results are compatible with membrane leakage, which may be promoted by oxidation of the lipid membrane components [98,106], from which TBARS are biomarkers. ALP and GGT activities were also found to be increased; since their synthesis increases and their excretion is blocked in case of intra or extrahepatic obstruction, both are cholestasis biomarkers [103,104]. Consequently, tramadol- and tapentadol-induced hepatotoxicity involve hepatocellular and hepatobiliary injury. Regarding ALT activity, the increase obtained upon a 14-day administration, at all doses, approximately doubled that of a single administration of 50 mg/kg tramadol or tapentadol [98]; similarly, while ALP activity did not change significantly as a result of an acute treatment [98], it increased at almost all opioid doses following repeated administration. Therefore, we might anticipate that hepatocyte and hepatobiliary damage is cumulative. 

### 3.3. Repeated Exposure to Tramadol and Tapentadol Compromises Liver Synthesis

As seen in our acute exposure assays, serum BuChE activity decreased at all tramadol and tapentadol doses when administered repeatedly (Figure 2). BuChe has been described as a sensitive marker of liver parenchyma cell inflammation and damage in patients with chronic hepatitis, with lower serum levels indicating higher severity of liver fibrosis [104,107]. However, as previously discussed [98], decreased BuChE activity might result from opioid-induced inhibition, besides defective BuChE hepatic synthesis [98,104,107]. Since our liver histopathological analysis evidences fibrous tissue deposition, but no signs of marked fibrosis (Figure 9), reduced BuChE activity may reflect both phenomena and ultimately indicate the potential for progression to fibrosis.

The metabolic impact of the exposure to both opioids has also been studied. While serum α-1-acid glycoprotein levels did not change significantly (Figure 2), serum complement C3 and C4 (Figure 2), albumin and urea (Figure 3) concentrations decreased upon exposure to tramadol and tapentadol, at almost all doses. In the case of urea, its urinary output is also lower at 50 mg/kg tapentadol, probably because of its decreased production (Figure 5). Urea concentrations had already been found to be diminished in our previous acute administration assays [98], though serum levels had decreased significantly for the 50 mg/kg tramadol/tapentadol only. In this context, the quantification of serum ammonia would provide additional information. In turn, albumin levels were found to be decreased in a tramadol-induced fatal overdose with liver failure [59]. Also, decreased serum albumin and total proteins were reported in opium-addicted diabetic males [108], as well as upon repeated intramuscular administration of 40 mg/kg tramadol [86]. Such results show that liver synthetic function is impaired, since these analytes are exclusively or primarily produced by this organ [103]. Indeed, liver disease is associated with hypocomplementemia: it is due to decreased C3 and C4 synthesis in fulminant hepatic failure, whilst in chronic active hepatitis it results from the formation of immune complexes and consequent complement activation [109]. A possible explanation for the fact that α-1-acid glycoprotein was the only protein whose levels did not change is its considerably longer half-life (164.8 h in rats) [110,111], when compared with those of complement C3 and C4 (46-70 h in humans) [112], albumin (2.6 h in rats) [113] and urea (5 h in rats) [114]. Therefore, due to its longer half-life, α-1-acid glycoprotein is not as useful as complement proteins as a biomarker to evaluate acute or subacute toxic exposures.

### 3.4. Repeated Exposure to Tramadol and Tapentadol Affects Lipid Profile, Correlating with Hepatobiliary Commitment and Lipid Deposition

The lipid profile is also altered, with increased triglyceride levels at all tapentadol doses, increased total cholesterol at 50 mg/kg tramadol, and increased LDL cholesterol at all doses from both opioids (Figure 3). No significant changes were identified regarding HDL cholesterol (Figure 3). When compared with our previous acute exposure results [98], the increase in triglyceride and LDL cholesterol levels is now extensible to more doses, suggesting that the derangement in lipid metabolism is also cumulative. While human studies are inconsistent, animal assays with opium, morphine, heroin and tramadol have proven to be more conclusive towards a deleterious impact of opioid use on lipid profile and dyslipidemia [115,116,117]. Although El-Gaarafawi, Youssef and Othman and respective colleagues have reported decreased serum cholesterol, triglycerides and lipid-derived hormones [75,81,90], Ezzeldin and co-workers have reported increased cholesterol [77]. Also, while assays with healthy, hypercholesterolemic and diabetic rodents, mostly comprising oral opium administration for 1 to 3 months, have shown no major effects on serum lipid parameters [118,119,120], others have reported increased serum triglycerides, total and LDL cholesterol, and decreased HDL cholesterol [121,122,123,124]. In this context, various mechanisms have been proposed to explain the action of opium consumption on blood and tissue lipids [115,116]. Short-term effects may be justified by increased lipolysis in adipose tissue, increased lipogenesis in liver [125] and decreased biliary cholesterol excretion [121], the latter being corroborated by our ALP and GGT results. In turn, long-term outcomes may derive from liver damage and insufficient lipid turnover [82,91], decreased hepatic LDL clearance and increased hepatic triglyceride synthesis [126], among others [115]. Overall, these mechanisms explain the most frequent serum lipid findings in animal studies – unchanged or increased triglycerides, total and LDL cholesterol, as well as unchanged or decreased HDL cholesterol [119,127]—which are substantiated in our own study. Interestingly, since cholesterol has a prominent role on the central nervous system and on synaptic plasticity [128], a relationship with drug addiction might be remotely implied and remains a subject for further scrutiny. 

### 3.5. Repeated Exposure to Tramadol and Tapentadol Affects Iron Metabolism, Correlating with Oxidative Stress, Cellular Damage, Inflammation and Steatosis 

Tramadol and tapentadol repeated administration also impacted iron metabolism, as increased serum iron levels were also found in most opioid experimental groups (Figure 4). In line with our results, a comparative study between non-insulin-dependent diabetes mellitus opium-addicts and non-addicts showed increased iron levels in addicted males [108]. Indeed, iron is implicated in dopamine synthesis and monoamine metabolism, having been shown to accumulate in specific brain regions in chronic cocaine use [129,130]. It is noteworthy that free iron may generate reactive oxygen species (ROS), such as the powerful hydroxyl radical, via Fenton chemistry—thereby worsening inflammation—and is profibrogenic [131,132]. Alterations in serum iron levels prompted the investigation of iron metabolism-related parameters (Figure 4). Serum ferritin, haptoglobin and HO-1 levels increased upon tramadol and tapentadol treatment; transferrin decreased upon tramadol exposure, while hepcidin decreased for tramadol highest dose and tapentadol lowest dose. In turn, B2M concentrations decreased at all opioid doses (Figure 4).

Ferritin is a positive acute phase protein (APP) [133], whose synthesis increases in case of oxidative stress and inflammation, or due to increased iron uptake by hepatocytes [134,135]. Since it is a safe form of iron storage, its serum form is argued to arise from damaged cells, thus representing a cellular damage marker [135,136]. Serum ferritin levels correlate with serum markers of hydroxyl radical formation, including MDA [136]. In this context, it has been hypothesized that, unlike its intracellular form, serum ferritin releases iron, which induces hydroxyl radical formation and consequent oxidative stress [136]. Therefore, increased serum ferritin levels are consistent with elevated serum iron concentrations, as well as with our results regarding oxidative stress and hepatocyte damage.

Hepcidin, in turn, is a hormone that binds ferroportin and elicits its internalization and degradation, preventing iron release from macrophages, hepatocytes and enterocytes [131,135,137,138]. Though hepcidin levels did not change significantly at all opioid doses, its decrease at tramadol and tapentadol highest and lowest doses, respectively, might also account, at least in part, for increased serum iron availability. 

Serum B2M is a small protein that non-covalently binds to the other polypeptide chain to form major histocompatibility complex (MHC) class I or MHC I-like structures, including human hemochromatosis protein (HFE) [139,140,141,142]. Since it is filtered in the glomeruli and massively reabsorbed in the proximal tubules, low serum and high urine concentrations indicate renal tubular disease [139,140,141,142,143,144,145]. Although this condition could be hypothesized in view of decreased serum B2M at all opioid doses, increases in its urine levels were not statistically significant (results not shown). However, an association between B2M, hepcidin and iron circulating levels might be postulated. B2M interacts with HFE in order to allow its surface expression; this, in turn, interacts with hepcidin, which prevents intracellular iron release. Thus, B2M influences iron uptake and efflux mediated by HFE and hepcidin, respectively [138]. Indeed, B2M-deficient mice present iron overload and hemochromatosis, whose pathogenesis likely involves other B2M-interacting protein(s) [138,139,146,147]. Therefore, the decreases in B2M and hepcidin levels might be correlated and, eventually, lead to both serum and liver iron accumulation. High hepatic iron content has been suggested as a steatosis causative agent, given iron involvement in oxidative stress and LPO, with consequent lipid biosynthesis and accumulation [147].

Transferrin, an iron transport protein [135], was found to decrease upon repeated administration of Wistar rats with 25 and 50 mg/kg tramadol (Figure 4). It is a negative APP [135], suggesting that tramadol treatment might be particularly inflammatory. Indeed, transferrin is lower in patients with cirrhosis, fatty liver disease and impaired synthetic function; low transferrin and high ferritin, a combination that, in the present study, is seen for tramadol, may indicate inflammation [148]. Toxic nontransferrin-bound iron is uptaken by hepatocytes, causing their overload; hepatocellular impairment then follows, decreasing hepcidin production and leading to uncontrolled iron release from cells [148], which is compatible with our results. Thus, it is arguable whether increased serum iron levels are a driver or a consequence of liver disease [148].

Serum HO-1 levels were found to be increased upon exposure to 50 mg/kg tramadol and tapentadol (Figure 4), while its gene expression in liver and kidney significantly increased upon exposure to 50 mg/kg tapentadol only (Figure 6). HO-1 is an inducible isoform of heme oxygenase whose expression is increased by several stimuli, including drugs, cytokines and ROS [131,149,150,151,152,153,154,155]. HO-1 catalyzes the conversion of heme into biliverdin, carbon monoxide and iron, which collectively provide its antioxidant, antiapoptotic, anti-inflammatory, anti-fibrotic and tissue repair properties [131,132,149,150,151,156,157,158,159]. Oxidized LDLs have been suggested to induce HO-1 expression in endothelial cells, smooth muscle cells and macrophages [149]. Although we did not specifically quantify oxidized LDLs, we have shown increased serum LDL cholesterol and increased LPO in liver and kidney cells, for which we might also hypothesize LDL oxidation–and, thus, a correlation with HO-1 induction. Interestingly, and similarly to our results, HO-1 expression has been shown to be increased in non-alcoholic steatohepatitis and to reflect the severity of the disease, with a significant correlation with ferritin and LPO [131,149]. Hence, in our study, HO-1 overexpression might be a response to increased oxidative stress (including eventual LDL oxidation), an attempt to curtain fibrosis, correlated with hepatic lipid deposition and ferritin increase and, ultimately, with some extent of liver and kidney disease. Indeed, lack of HO-1 induction has been associated with oxidative damage and hepatic and renal iron accumulation, as well as with chronic inflammatory states [131,156,157]. Its up-regulation has been reported in experimental models of hepatic porphyria, fibrosis, cirrhosis, among other liver injury situations [151,160], as well as in several renal disorders, including acute kidney failure, acute glomerulonephritis and other glomerular, tubular, interstitial and vascular diseases, having been suggested as a candidate disease biomarker [157,158].

Haptoglobin, a glycoprotein mostly synthetized in the liver, stoichiometrically combines with hemoglobin, participating in its turnover and clearance by the mononuclear phagocyte system, also mainly in the liver; thus, it contributes to iron homeostasis and prevents its oxidative activity [133,161,162,163]. Increased haptoglobin levels are found in patients with obstructive biliary disease, where a correlation between its levels and ALP has been identified, suggesting that a higher level in obstruction might be related to biliary retention [161,162]. Haptoglobin also reduces hemoglobin loss through glomeruli, preventing renal iron loading during aging and following acute plasma heme-protein overload [163]. In addition, since it is a major or moderate APP (depending on the species), showing anti-inflammatory properties and binding to integrins on leukocytes, its increase is also a response to inflammation [133]. Therefore, in our study, increased haptoglobin levels might be due to a combined status of biliary obstruction (already suggested by augmented GGT, ALP, total cholesterol and LDL cholesterol) and inflammation, as well as to a possible attempt to minimize renal iron overload.

### 3.6. Repeated Exposure to Tramadol and Tapentadol Compromises Kidney Glomerular and Tubular Functions

In turn, nephrotoxicity is reported as a consequence of opioid exposure [164]. Rhabdomyolysis, secondary amyloidosis, membranous nephropathy, nephrotic syndrome, acute glomerulonephritis, focal and segmental glomerulosclerosis due to deposition of immune complexes, progressive chronic renal failure and tubular epithelial cell degeneration have been observed in chronic heroin, morphine and methadone users [164,165,166,167]. Moreover, there is an association between cholestasis—suggested by some of our results—kidney tubular changes and nephrotoxicity, though the exact underlying mechanisms are not known [166]. Elevated levels of opioid agonists may exert deleterious effects through oxidative stress, nitric oxide (NO) overproduction, apoptosis and vascular endothelial dysfunction [166]. ROS induce LPO in renal arterial endothelium, mesangial and renal tubular cells, causing renal failure [166].

In the present study, the alterations in serum uric acid were not statistically significant, as well as those in urinary total protein levels (Figure 5), the latter opposing the evidences of proteinuria seen in our acute exposure studies [98]. Nevertheless, all other renal function biomarkers assayed are compatible with kidney damage.

Serum cystatin C, regarded as a more accurate and sensitive marker of early kidney dysfunction than serum creatinine, increased at tapentadol highest dose. This might reflect a lower glomerular filtration rate [98,142,168,169,170]. Although urinary levels did not change significantly (results not shown), its mere detection in urine samples reflects proximal tubular injury, since cystatin C is reabsorbed and catabolized by tubular cells, with no tubular secretion [142,170].

Figure 5 also evidences that microalbuminuria (i.e., moderate increases in urine albumin) occurs at all tapentadol doses. Albuminuria may derive from increased glomerular permeability due to endothelial cell, basement membrane or podocyte dysfunction, as well as to inhibited proximal tubule reabsorption [171]. Given albumin role as a fatty acid transporter and that proteinuric kidneys preferentially lose albumin with low fatty acid content, there is a progressive retention of albumin with high fatty acid content, leading to serum fatty acid accumulation and their limited uptake by skeletal muscle, heart and adipose tissue [172,173]. This correlates well with increased serum triglycerides—since they are composed of fatty acids—which were compatibly observed at all tapentadol doses (Figure 3).

Urinary creatinine levels also decreased at all tapentadol doses, which may reflect decreased glomerular filtration; indeed, the degree of urinary creatinine decline has been associated with faster renal disease progression and poorer outcomes [168,174]. Conversely, several in vivo studies, concerning oral and intramuscular tramadol acute and chronic administration to rats, mice and rabbits, at doses ranging from 10 to 300 mg/kg, have led to increased serum creatinine concentrations [72,74,75,76,77,78,79,81,83]. The same trend was found among tramadol abusers [102].

Serum amylase activity is also elevated at all opioid doses, which might be associated with renal impairment, since amylase enters urine primarily via glomerular filtration, with partial tubular reabsorption [175,176,177]. Indeed, altered amylase clearance might arise from increased glomerular permeability and tubular dysfunction, both in acute and chronic kidney disease [176,177]. Hyperamylasemia may occur in other conditions, such as acute pancreatitis, which was not investigated in the present work; however, the elevation seen in renal insufficiency is rarely greater than 2 times the upper reference limit [178,179], which is compatible with the depicted in Figure 5. Liver disease might also account for increased serum amylase levels, since a large proportion of the circulating enzyme is cleared by the mononuclear phagocyte system and subsequent removal through bile [180,181]. In this context, a combination of renal impairment with biliary obstruction, whose presence has already been suggested by our results, may contribute to elevated serum amylase.

Urinary NAG activity increased at 50 mg/kg tramadol and 25 and 50 mg/kg tapentadol. NAG is a lysosomal enzyme of the proximal tubule epithelial cells; due to its large molecular weight, it is not filtered through the glomerulus, and is neither absorbed nor secreted by renal tubules. Unlike other renal function biomarkers that are filtered through the glomerulus, increased urine levels of NAG, deriving exclusively from tubule cells, specifically reflect proximal tubule dysfunction [142,182,183,184,185,186]. NAG has been suggested as a more sensitive biomarker of early nephropathy than albuminuria [185,186]. Interestingly, increased urinary NAG activity, as well as renal morphologic changes, were found in cholestatic rats and reversed by naltrexone treatment, suggesting the involvement of endogenous opioids in cholestatic nephrotoxicity [183]. Since our data are compatible with biliary obstruction, the hypothesis of exogenous opioid-induced cholestatic nephrotoxicity could be considered.

### 3.7. Repeated Exposure to Tramadol and Tapentadol Alters Hepato-Renal Toxicity Biomarker Gene Expression, Correlating with Metabolic Changes, Cell Toxicity and Glomerular Dysfunction

Concerning liver expression of hepatotoxicity biomarker genes (Figure 6a), Aldoa (encoding for fructose-bisphosphate aldolase A, a glycolytic enzyme) significantly increased upon tramadol exposure, as previously seen in serum from patients with fulminant hepatitis [187] and drug-induced liver injury [188], as well as in liver tissue from animal models acutely and sub-acutely exposed to different xenobiotics [189,190,191,192]. Aldoa upregulation has also been reported in cirrhotic and hepatocellular carcinoma livers [193,194], confirming the high glycolytic phenotype as a typical feature of both precancerous and cancerous lesions. Enhanced glucose oxidation (and inherent glycogen mobilization) may represent a metabolic response to tramadol-induced stress.

Apex1, in turn, encodes for apurinic/apyrimidinic endonuclease 1, an enzyme involved in base excision repair and a regulator of gene expression as a redox co-activator of different transcription factors [195]. Apex1 up-regulation was observed in liver tissue from drug-treated rodents, since its expression is induced by ROS as a defense mechanism against genomic instability [160,191,192,195]. Since Apex1 gene expression did not change significantly in our study, it might be hypothesized that, in the conditions that were assayed, genotoxicity is not a predominant hepatotoxicity mechanism, or that repair mechanisms are not yet being recruited. Additional studies are needed in order to confirm these hypotheses.

Cd36 encodes for cluster of differentiation 36/fatty acid translocase, showing ability to bind oxidized LDL, long chain fatty acids, phospholipids and collagen [196,197,198]. Increased expression in hepatocytes is associated with augmented fatty acid uptake, triglyceride accumulation and, thus, hepatic fibrogenesis, steatosis and non-alcoholic fatty liver disease [196,197,198]. Furthermore, in several mouse strains, it has been identified as the gene having highest correlation with fatty liver, and its disruption has been shown to protect against systemic inflammation and insulin resistance [196,198]. Thus, Cd36 overexpression upon exposure to 50 mg/kg tramadol might be correlated with the high total and LDL-cholesterol serum levels, as well as with a higher profibrogenic potential.

Lpl, in turn, encodes for lipoprotein lipase, an endothelium-anchored enzyme that catalyzes the hydrolysis of triglycerides from chylomicrons and very low density lipoproteins (VLDL) into free fatty acids, enabling their uptake by extrahepatic tissues [199,200,201]. The decrease in Lpl expression, as seen upon exposure to 50 mg/kg tramadol and tapentadol, might therefore be associated with higher serum triglyceride levels—which is observed in tapentadol groups (Figure 3)—and higher serum cholesterol levels—as seen in the 50 mg/kg tramadol group (Figure 3)—as these lipids are transported in the form of lipoproteins. Indeed, lipid disorders frequently accompany liver disease, with increased hepatic secretion of VLDL particles due to increased concentration of free fatty acids and glucose, and decreased VLDL clearance due to reduced activity of lipoprotein lipase [201]. 

Regarding the nephrotoxicity biomarker gene panel, Angptl4 kidney expression was found to be upregulated in tapentadol-treated rats (Figure 6b). Angptl4, angiopoietin-like 4 protein, is secreted from podocytes, having been implicated in processes as diverse as glucose and energy homeostasis, angiogenesis and vascular permeability, inflammation, tumorigenesis, cell differentiation, wound healing and redox regulation [202,203,204]. It induces morphological and clinical manifestations of human minimal change disease and is being increasingly recognized as a contributor to proteinuria in experimental diabetic nephropathy [152,172,173,205]. However, one of its most studied roles is as a regulator of lipid metabolism, having been shown to modulate both intracellular and extracellular lipolysis [206], and linked to lipoprotein lipase inhibition and hypertriglyceridemia in nephrotic syndrome [173,202,204,206,207,208], which correlates well with the increased serum triglyceride levels (Figure 3) and decreased liver Lpl expression (Figure 6a) observed in tapentadol groups. 

Gamt, in turn, encodes for guanidinoacetate *N*-methyltransferase, the enzyme that catalyzes the last step of creatine biosynthesis [209]. Its gene expression has been shown to be downregulated in kidneys from tramadol- and tapentadol-administered rats (Figure 6b). In line with our results, Gamt inhibition and down-regulation have been reported following drug-induced nephrotoxicity and suggested as a result of toxicity progression and biochemical feedback mechanisms to compensate for altered creatinine clearance, since creatinine is a product of creatine [153,154,209,210]. Lower Gamt activity leads to decreased creatine synthesis and precursor buildup; while the former ultimately compromises the creatine/phosphocreatine energy buffer system, the latter has been associated with cell toxicity through a number of mechanisms [211].

Nphs2 encodes for podocin, a slit diaphragm protein that acts as a structural scaffold in podocyte foot processes and interacts with other slit diaphragm proteins to facilitate anti-apoptotic signaling events. It is essential for the establishment and maintenance of the glomerular filtration barrier [212,213,214,215,216], having been found to be downregulated in lupus nephritis, pediatric nephrotic syndrome and focal segmental glomerulosclerosis [217]. Indeed, loss of podocin, as well as inactivating mutations on its gene, are associated with glomerular lesions (including mesangial proliferation), glomerulosclerosis, albuminuria, hypercholesterolemia, hypertension, and renal failure, which characterize nephrotic syndrome [212,213,214,215,218]. Thus, since Nphs2 gene expression is significantly decreased in tramadol-treated rats (Figure 6b), glomerular injury might be anticipated. Such hypothesis is consistent with the results concerning other glomerular function biomarkers, such as serum amylase (Figure 5).

### 3.8. Repeated Exposure to Tramadol and Tapentadol Causes Liver and Kidney Histopathological Changes, Correlating with Metabolic and Gene Expression Alterations

The hepatic and renal effects of the repeated administration of tramadol and tapentadol clinical doses were also studied at the histological level, reinforcing the results from our previous acute exposure assays to the same doses [98]. In addition, such results also have forensic significance, since acute liver failure, extensive fulminant necrosis, marked steatosis, congestion and enlargement have been reported upon lethal intoxication with both tramadol [59,219,220,221] and tapentadol [54].

Regarding liver, sinusoidal dilatation was a recurrent finding at all opioid doses, being more profuse on tapentadol groups (Figure 7, Figure 8 and Figure 9). Such results had already been reported in acute to sub-chronic rat exposure assays to tramadol doses ranging from 12.5 to 300 mg/kg [71,77,78,92,93,94]. Mononuclear cell inflammatory infiltrates were another seemingly dose-dependent finding for both opioids (Figure 7 and Figure 8), which is also consistent with similar exposure studies, mostly sub-chronic and chronic [76,78,85,88,92,93,94,95]. Signs of cellular degeneration, including nuclei fragmentation and poor definition, were increasingly apparent along with tramadol dose, while they were observed at all tapentadol doses, on whose slides hypopigmented areas could also be seen (Figure 7 and Figure 8). Indeed, related cellular and tissue alterations, comprising necrosis, apoptosis, hydropic degeneration, karyolitic and pyknotic nuclei, cytolysis, tissue disorganization and loss of architecture, were reported in analogous studies using mainly tramadol, but also heroin, nalbuphine and morphine [71,73,76,77,78,79,81,84,85,87,88,90,92,93,94,95]. In turn, vascular congestion, with erythrocyte extravasation, was unique to tapentadol exposure (Figure 7), as seen in our previous acute exposure assays [98]. In this context, there are reports of congestion, dilated blood vessels, hemorrhage and stagnant blood upon exposure to 3 to 300 mg/kg tramadol—but also in studies concerning opioids such as morphine, heroin and nalbuphine—for periods ranging from acute to chronic [71,73,76,77,78,79,84,85,87,88,92,93,94,95]. Hepatocyte vacuolization and microsteatosis were also consistent observations (Figure 7, Figure 8 and Figure 9), again in line with comparable studies [74,76,79,84,85,92,93,94]. As already discussed, such evidence might be correlated with the derangement of lipid metabolism, increased iron levels and elevated Cd36 gene expression. In turn, PAS staining evidenced lower liver glycogen accumulation in experimental groups (Figure 8), in line with the observed in our previous acute exposure studies [98] and upon a 20-day period of daily oral administration of 40 mg/kg tramadol to rats [88]. Though glycogen depletion may indeed be due to the 24h-fasting that preceded rat sacrifice, the controls still present denser glycogen masses, showing that glycogenolysis might be a compensatory mechanism to cope with opioid-induced metabolic stress [98]. Enhanced glycolysis, corroborated by Aldoa gene overexpression in tramadol-treated rats (Figure 6a), might be a downstream event. Finally, Masson’s trichrome staining revealed fibrous tissue accumulation between hepatocytes, which was particularly evident on liver specimens from tapentadol-exposed rats (Figure 9). On one hand, such observation may be interpreted as a sign of revascularization, a possible response to liver injury, and is supported by studies concerning mostly sub-chronic exposure to tramadol therapeutic and supratherapeutic doses [76,77,84,88,94,95]. On the other hand, increased collagen fibers were suggested to be the result of ROS deleterious action either on collagen itself or on enzymes involved in its metabolism [222], which may represent an additional explanation. Moreover, hepatic microsteatosis and fibrosis might be correlated with increased liver iron content [131,132,147], which is also hypothesized in this paper. 

Interestingly, histopathological studies performed upon exposure to tramadol doses up to 300 mg/kg and for periods up to 150 days do also report bile duct proliferation and hyperplasia—which are mainly associated with biliary disorders [223,224]—as well as cholestatic hepatitis [76,78,94,95]. Also, non-fatal cases of tramadol poisoning report hepatobiliary dysfunction [59]. Although, in our study, this was not a valuable finding from the histopathological point of view, our biochemical results—increased GGT, ALP, total cholesterol, LDL cholesterol and haptoglobin—are consistent with biliary obstruction. Thus, it might be hypothesized that dose and/or exposure time increments lead to the accumulation of histological evidence of biliary disease.

Concerning kidney histopathological study, disorganized and poorly contoured tubules, as well as increased Bowman’s spaces, were omnipresent findings on all opioid group slides, and cell swelling was observed at all tramadol doses (Figure 10). Such observations are in line with those from similar studies, which report tubular endothelial cell degeneration, vacuolization, swelling and even necrosis [71,74,76,77,78,81,88,95]. A case report of a fatal intoxication by tapentadol does also mention kidney cell autolytic changes [54]. In turn, while glomerular disorganization and vacuolization were patent at all tapentadol doses, they became increasingly evident along with tramadol dose (Figure 10). In this context, several analogous studies report glomerular atrophy, with collapsed tufts [76,81,88,95]. It is also noteworthy that mononuclear cell infiltrates were observed on tramadol slides only, irrespectively of the dose considered. Studies concerning tramadol oral, intramuscular and i.p. administration to rats and sheep, at doses ranging from 5 to 300 mg/kg, refer similar findings [71,76,78,81,88,95]. Some of these studies also report hemorrhage, congestion, inter-tubular blood vessel dilatation and thickening, and even renal cast formation/mineralization in corticomedullary tubules [71,76,77,81,95,96], although we did not find relevant signs of them. In addition, Elkhateeb and co-workers reported an increase in collagen fibers in rat kidney samples upon a 30-day exposure period to 30 mg/kg tramadol [76], for which it would be interesting to assess whether a shorter, 14-day exposure period to a similar dose (25 mg/kg) and/or to a higher dose (50 mg/kg) produces similar results. However, we did not perform Masson’s trichrome staining with kidney specimens; thus, that may only be hypothesized.

Taken together, the results of the present work offer additional insights to our previous studies addressing liver, kidney, heart, lung and brain cortex toxicity following an acute exposure to the same tramadol and tapentadol doses [98,99]. Our biochemical and histological analysis shows that hepatic and renal alterations, at the metabolic and histopathological levels, occur and accumulate subsequently to longer periods of administration than that previously assayed, but shorter than those implemented in most comparable repeated administration studies, and for lower tramadol and tapentadol doses.

## 4. Materials and Methods

### 4.1. Chemicals

Tramadol hydrochloride was obtained from Sigma-Aldrich (St. Louis, MO, USA), while tapentadol hydrochloride was provided by Deltaclon (Madrid, Spain). Both compounds were dissolved and diluted in saline (0.9 g/L (*w/v*) NaCl) immediately prior to administration. Sodium thiopental was obtained from B. Braun Medical (Queluz de Baixo, Portugal). All other chemicals were commercial preparations of the highest available degree of purity.

### 4.2. Experimental Models and Animal Handling

42 male Wistar rats, aged 8 weeks and weighing 250–300 g, were provided by the i3S animal facility (Porto, Portugal). All animals were housed in acrylic cages with wood chips and paper towels as enrichment items, under controlled standard laboratory conditions (22 ± 2 °C, 50–60% humidity, 12/12 h light/dark cycles). Rats were given *ad libitum* access to tap water and rat chow (standard short and middle period maintenance formula for rodents, reference 4RF21, Mucedola/Ultragene (Milan, Italy), as well as a quarantine period of at least one week before experimental assays.

Animal experimentation complied with the European Council Directive (2010/63/EU) guidelines, transposed into the Portuguese law (Decree-Law no. 113/2013, 7th August). All assays were also approved by the Ethics Committee of CESPU, Institute of Research and Advanced Training in Health Sciences and Technologies (IINFACTS), Gandra, PRD, Portugal (processes no. PI4AC 2017, PI4AC 2018 and PI-3RL 2019), and complied with the National Ethics Council for the Life Sciences (CNECV) guidelines.

### 4.3. Experimental Design and Drug Treatment

Wistar rats were randomly assigned to seven groups, composed of six animals each. The sample size/number of animals per group was determined through the G*Power software, version 3.1.9.6 (Heinrich-Heine-Universität Düsseldorf, Düsseldorf, Germany), assuming a significance level of 0.05, an 80% power and effect size values adjusted accordingly with the biochemical parameters to be analyzed (based on literature and on the previous experience of the team in similar analyses).

Drug treatment consisted of daily i.p. injections of 1 mL-units, using saline (0.9% (*w/v*) NaCl) as vehicle, at the same time every day, for 14 consecutive days. Group 1 (control group) received saline administrations, groups 2, 3 and 4 received 10, 25 and 50 mg/kg tramadol, respectively, while groups 5, 6 and 7 received 10, 25 and 50 mg/kg tapentadol, respectively. 

Rat doses were determined by converting the human dose into the animal equivalent dose (AED), using a body surface area correction factor (*K_m_*) of 6.2 and the following formula, assuming an average 60 kg-human: AED (mg/kg) = human dose (mg/kg) × *K_m_* ratio [225,226]. In order to establish opioid doses for rat administration, their median lethal dose (LD_50_) for rats [227], concentrations reported in intoxications [56], and tramadol and tapentadol maximum recommended daily doses for humans [2,24,227,228] were considered. Except for specific pathological conditions or other clinically relevant situations, the standard tramadol dose for a 60-kg patient is 50–100 mg (1.67 mg/kg/day) three to four times a day, totaling a maximum recommended daily dose of 400 mg [1,227]. In turn, tapentadol maximum recommended daily dose is reported as 600–700 mg/day [1,24,228]. The 1.67 mg/kg/day standard, corresponding to a 100 mg-dose, is thus equivalent to 10.35 mg/kg (when multiplied by 6.2). Accordingly, 10 mg/kg corresponds to an effective, analgesic 100 mg-dose; 25 and 50 mg/kg are equivalent to an intermediate and the maximum recommended daily dose, respectively, considering a 60 kg-adult [98,99].

Immediately after the last administration, rats were placed in metabolic cages and given unlimited access to tap water, but no food, for the remaining 24 h. Animals were kept under monitoring throughout this period, upon which they were sacrificed.

### 4.4. Collection and Processing of Biological Samples

Urine samples were collected from each animal, into an ice-cold container, during the last 24 h-exposure period. Samples were processed through centrifugation at 3000× *g*, 4 °C, for 10 min, to remove any debris. Animals were sacrificed by means of anesthetic procedures (i.p. injection with 60 mg/kg sodium thiopental, using saline as vehicle). Blood samples were drawn with a hypodermic heparinized needle, through cardiac puncture, and further submitted to centrifugation at 3000× *g*, 4 °C, for 10 min, to obtain serum. Samples were then aliquoted and stored (−80 °C) for further biochemical analysis.

Livers and kidneys were surgically collected, dried with gauze, weighed on an analytical balance, and further processed. One portion of each organ was homogenized in an Ultra-Turrax^®^ (IKA^®^, Staufen, Germany) in 1:4 (*w*/*v*) ice-cold 50 mM phosphate buffer (KH_2_PO_4_ + Na_2_HPO_4_·H_2_O), pH 7.4. The respective supernatants were obtained through centrifugation at 4000× *g*, 4 °C, for 10 min. The aliquots thus obtained, as well as the remaining intact portions of the organs, were stored at −80 °C, regarding subsequent analysis.

#### 4.4.1. Quantification of Oxidative Stress Parameters

Oxidative stress was assessed, in liver and kidney homogenates, as the degree of LPO and protein oxidation, through the quantification of TBARS and protein carbonyl groups (ketones and aldehydes), respectively. The total antioxidant capacity was also determined in the same samples.

Total protein content was determined through the Pierce™ BCA Protein Assay Kit (Thermo Scientific, Rockford, IL, USA), according to the manufacturer’s microplate procedure.

Perchloric acid was added to liver and kidney homogenates to a final concentration of 5% (*w*/*v*), to precipitate proteins. Samples were centrifuged at 13,000× *g*, 4 °C, for 10 min, with both pellets and supernatants being stored at −80 °C for subsequent analysis. LPO quantification was performed in supernatants, through the TBARS method reported by Buege et al. [229]. Results were expressed in terms of nanomoles of MDA equivalents per milligram of protein.

In turn, carbonyl groups were quantified in protein pellets, according to Levine et al. [230]. Results were expressed as nanomoles of DNPH incorporated per milligram of protein.

The total antioxidant capacity was determined with the Total Antioxidant Capacity Assay Kit (Sigma-Aldrich), following the manufacturer’s instructions. Liver homogenates were diluted 20-fold, while kidney samples were used directly. Results were expressed in terms of mM of antioxidants (Trolox equivalents) per milligram of protein.

#### 4.4.2. Quantification of Biochemical Parameters in Serum and Urine Samples

Albumin, ALP, ALT, amylase, AST, α-1-acid glycoprotein, BuChE, total cholesterol, HDL cholesterol, LDL cholesterol, complement C3 and C4, GGT, iron, ferritin, haptoglobin, transferrin, total proteins, triglycerides and uric acid were quantified in serum samples, while urine proteins, creatinine, microalbumin and NAG were determined in urine samples. Cystatin C, B2M and urea were determined both in serum and urine samples. Unless otherwise stated, biochemical parameters were quantified in an automated analyzer (Prestige 24i, Tokyo Boeki, Tokyo, Japan), according to the manufacturer’s instructions, as previously described [97,98,99,231], and using undiluted samples. Calibrations were appropriately performed for each parameter, with two appropriate calibrators, in order to plot 5-point standard curves. Quality controls were also included. All automated analyzer reagents were supplied by Cormay PZ (Warsaw, Poland), except for those concerning B2M, which were purchased from Spinreact (Barcelona, Spain). 

NAG activity was quantified with the NAG assay (Diazyme, Poway, CA, USA), according to the manufacturer’s directions. Urine proteins were determined through the microplate procedure of Pierce™ BCA Protein Assay Kit (Thermo Scientific), upon removal of interfering substances according to Yalamati and co-authors [232] and a 6-fold sample dilution in 0.5 N NaOH.

Enzyme activities were determined as U/L, while biochemical parameters were retrieved as mg/dL, except for albumin (g/dL), cystatin C, B2M and microalbumin (mg/L), ferritin (μg/L), iron (μg/dL) and serum and urine proteins (g/L).

In turn, HO-1 and hepcidin were determined in serum samples, through enzyme-linked immunosorbent assay (ELISA), using the HO-1 (rat) ELISA kit (Enzo Life Sciences, Farmingdale, NY, USA) and the Rat Hepcidin (Hepc) ELISA kit (Abbexa, Cambridge, UK), respectively, according to the manufacturers’ specifications. For HO-1 quantification, samples were diluted 10-fold with sample diluent, while undiluted samples were used for hepcidin analysis. ELISA results were retrieved as ng/mL (HO-1) or pg/mL (hepcidin).

#### 4.4.3. Gene Expression Analysis through qRT-PCR

Total RNA was isolated from liver and kidney samples using the NZYol reagent (NZYTech, Lisbon, Portugal), according to the manufacturer’s instructions concerning tissue samples. RNA integrity was assessed through 1.4% (*w*/*v*) agarose gel electrophoresis. RNA purity, regarding protein and organic compound contamination, was determined as the optical density (OD) OD_260 nm_/OD_280 nm_ and OD_260 nm_/OD_230 nm_ ratios, respectively (NanoDrop 2000 spectrophotometer, Thermo Scientific). Samples with OD_260 nm_/OD_280 nm_ and OD_260 nm_/OD_230 nm_ ratios ≥ 1.8 were selected for complementary DNA (cDNA) synthesis. 800 ng total RNA were converted into cDNA using the NZY First Strand cDNA Synthesis kit (NZYTech), according to the supplier’s instructions.

Gene expression was analyzed using the iQ™ SYBR^®^ Green Supermix (Bio-Rad Laboratories, Hercules, CA, USA), following the manufacturer’s directions. Each cDNA sample was diluted 10-fold in ultrapure water and analyzed in duplicate, totaling 12 replicates for each condition. Cd36, Aldoa, Apex1, Lpl, Angptl4, Hmox1, Nphs2 and Gamt genes were analyzed. 18S ribosomal RNA (18S rRNA) was used as housekeeping gene, for loading control purposes. Each amplification mixture totaled 25 µL, comprising 12.5 µL 2× iQ™ SYBR^®^ Green Supermix (Bio-Rad), 2 µL diluted cDNA, forward and reverse primers to a final concentration of 100 nM each, and 10 µL RNase-free water. The primers used for amplification (STABvida, Caparica, Portugal) are described in Table 1.

RNA template controls (RTC) and non-template controls (NTC) were included in each run. The qRT-PCR program was run in a C1000™ Thermal Cycler equipped with a CFX96™ Real-Time System, both from Bio-Rad Laboratories. The amplification program comprised an initial denaturation step at 95.0 °C for 3 min, and then 37–45 amplification cycles composed of a denaturation step at 94.0 °C for 20 s, an annealing step at 55.0 °C for 30 s, an extension step at 72.0 °C for 30 s and a plate read step. The number of amplification cycles used for the analysis of each gene is specified in Table 1. 

A melt curve was finally acquired between 65.0 and 95.0 °C, with 0.5 °C increments at every 5 s, followed by plate reads. Results were retrieved using the Bio-Rad CFX Manager software, version 3.1 (Bio-Rad Laboratories), and normalized against those of the control group. Relative changes in gene expression were determined through the Δ(Δ*C*t) algorithm.

#### 4.4.4. Liver and Kidney Histopathological Analysis

One portion of liver and kidney tissue from each animal was collected and fixed in 4% (*w/v*) formaldehyde, for 24 h at room temperature, for subsequent histological analysis. It was then submitted to standard dehydration and paraffin wax-embedding procedures, as previously described [242,243]. Three μm-sections were cut in a microtome (Shandon™ Finesse™ 325, Thermo Scientific) and adhered to glass slides. H&E, PAS and Masson’s trichrome staining procedures were performed with liver simples, while kidney samples were processed for H&E staining only. Slides were prepared through standard methods and observed under phase contrast microscopy, using 100× and 600× magnifications (Eclipse TE2000-U microscope, Nikon, Melville, NY, USA), coupled to a DXM1200F digital camera and controlled by Nikon ACT-1 software, version 2.70). Multiple microscope fields of observation were analyzed, and images were taken from representative ones.

### 4.5. Statistical Analysis

Results were expressed as means ± SD. Statistical data analysis was performed as an Analysis of Variance (ANOVA). Post-hoc analysis consisted of Dunnett’s multiple comparisons test. Probability values of *p* < 0.05 were considered as statistically significant. Graphic plotting and all statistical tests were performed using GraphPad Prism^®^ version 8.3.1 (GraphPad Software, LLC, San Diego, CA, USA). In all determinations, results were compared with those of the control group, injected with saline.

## 5. Conclusions

The increase in opioid prescription, use and abuse is accompanied by an increase in the number of adverse event reports. Although tramadol and tapentadol are known for their safety, having been designed to specifically address the drawbacks of their opioid peers, several adverse events and fatalities are being reported in the literature. Paradoxically, such phenomena are poorly documented at the molecular, biochemical, cellular, and histological levels. In this sense, our study attempts to fill some gaps regarding the mechanistic rationale underlying tramadol and tapentadol organ-specific toxicity. The novelty of the information applies most particularly to tapentadol, for which, owing to its shorter market history, there is fewer data available. In addition, our studies also represent an added value. In fact, while most toxicological information concerns full opioid receptor agonists, often at a supratherapeutic or overdose range, we provide comprehensive and comparative results for two partial agonists, administered at therapeutic doses. In this context, this is, to the best of our knowledge, the first in vivo study comparatively addressing tramadol and tapentadol toxicity upon repeated administration of clinically relevant doses. Furthermore, we have broadened the spectrum of parameters in relation to that studied in our previous acute assays, adding more biochemical/metabolic biomarkers, and including gene expression assays and additional histological staining methods.

In the present work, we demonstrate that a 14-day period of daily single administration of tramadol and tapentadol therapeutic doses induces hepato- and nephrotoxicity, as substantiated by changes in a panel of several biochemical, metabolic and histological parameters. Although some of the reported findings are exclusive to or more intense for tapentadol—a trend that had already been identified in our previous acute studies—the extension of the exposure tended to smooth the differences between the results from both opioids. Alterations proven to be more specific or more pronounced at the highest doses of one opioid, in our acute studies, were now shown to appear upon repeated administration of both opioids, and at lower doses. Oxidative stress biomarkers were augmented in both liver and kidney tissues, and liver synthetic function indicators, such as albumin, urea, BuChE and complement C3 and C4, were decreased upon exposure to both opioids. Alterations in the lipid profile, as well as in liver function tests such as ALT, AST, ALP and GGT, are strongly suggestive of hepatic dysfunction under the conditions assayed. Iron metabolism was also found to be deranged following exposure to both tramadol and tapentadol, as seen from the alterations in a panel including ferritin, haptoglobin and HO-1, among other related parameters. In turn, kidney function is also seemingly committed, and most prominently upon tapentadol treatment, as deduced from serum and urine alterations in parameters such as cystatin C, creatinine, microalbumin and NAG activity. Liver histopathological analysis revealed the presence of sinusoidal dilatation, inflammatory cell infiltrates, microsteatosis, glycogen depletion and cell degeneration. Accumulation of fibrous tissue was more evident following tapentadol treatment, to which erythrocyte extravasation was exclusive. Kidney histopathological findings comprised tubular and glomerular disorganization, as well as increased Bowman’s spaces, for both opioids, while mononuclear cell infiltrates and cell swelling were more apparent upon tramadol exposure. Gene expression assays have also identified quantitative changes in almost all liver and kidney toxicity biomarkers studied, upon exposure to either one or both opioids. Likewise, gene expression results correlate well with metabolic and histopathological results concerning, for instance, lipid and iron metabolism derangement, liver microsteatosis and kidney glomerular and tubular dysfunction. Therefore, rather than evident signs of cell death, repeated administration of tramadol or tapentadol at therapeutic doses elicits hepato- and nephrotoxicity mainly at the biochemical, metabolic and tissue organization levels.

Such results require reinforced attention from the scientific and clinical point of view, emphasizing the need for careful consideration of the maximum recommended daily doses, as well as for liver and kidney function monitoring when prescribing tramadol and tapentadol. Although tapentadol presents several advantages over tramadol, such as a more linear pharmacokinetics and properties that make it a better option for specific types of pain, it seemingly does not offer significant extra safety, as far as our endpoint results are concerned. Hence, the use of both tramadol and tapentadol should be carefully deliberated and monitored in patients with liver and/or kidney disease, particularly when more prolonged, subacute to chronic contexts of use are considered.

Additional studies, broadening the dose range assayed and extending the administration period, would further complement and clarify the results hereby presented, since they would shed light on the effects of chronic tramadol and tapentadol use. Immunohistochemistry assays, using appropriate toxicity/inflammation markers (e.g., tumor necrosis factor α (TNF-α), inducible NO synthase (iNOS), caveolin-1 (Cav-1) and pentraxin 3 (PTX3)), would also complement biochemical and histopathological analyses. Combined administration of tramadol/tapentadol with drugs that are often concomitantly used with them, such as selective serotonin reuptake inhibitors, tricyclic antidepressants, and monoamine oxidase inhibitors, would also be informative. Indeed, they would elucidate whether toxicological results are exacerbated by eventual drug-drug interactions and subsequent accumulation. The use of metabolites and/or opioid antagonists could also be considered in the experimental design. Also, to account for sex-dependent differences in drug metabolism, and considering that opioids are used in the treatment of sex-independent forms of pain, future studies should include female animals. Behavioral studies would also enlighten about abuse and dependence potential under comparable experimental settings.

## Figures and Tables

**Table 1:** Primer nucleotide sequences [233,234,235,236,237,238,239,240,241] and number of amplification cycles used for gene expression analysis of hepato- and nephrotoxicity biomarkers through quantitative Real-Time PCR (qRT-PCR).

Gene	Forward Primer (5′→3′)	Reverse Primer (5′→3′)	No. of Amplification Cycles	Reference
*Cd36* (Cluster of differentiation 36/fatty acid translocase)	AGGAAGTGGCAAAGAATAGCAG	ACAGACAGTGAAGGCTCAAAGA	37	[233]
*Aldoa* (Fructose-bisphosphate aldolase A)	ATGCCCCACCCATACCCAGCACT	AGCAGCAGTTGGCGGTAGAAGCG	37	[234]
*Apex1* (Apurinic/apyrimidinic endonuclease 1)	GAATGTGGATGGGCTTCGA	AAGATGTCTGGTGCTTCTTCCTTT	41	[235]
*Lpl* (Lipoprotein lipase)	CTTAAGTGGAAGAACGACTCCTACT	GTCATGGCATTTCACAAACACTGCA	41	[236]
*Angptl4* (Angiopoietin-like 4)	GCCGCTACTATCCACTAC	CCTGTTGCTCTGACTGTT	45	[237]
*Hmox1* (Heme oxygenase 1)	ACAGGGTGACAGAAGAGGCTAA	CTGTGAGGGACTCTGGTCTTTG	45	[238]
*Nphs2* (Podocin)	TGGAAGCTGAGGCACAAAGA	AGAATCTCAGCCGCCATCCT	38	[239]
*Gamt* (Guanidinoacetate *N*-methyltransferase)	ACTCATGCTTTCCGTTTGCT	AGGCACCTGAGTCTCCTCAA	38	[240]
*18S rRNA* (18S ribosomal RNA)	TTCGGAACTGAGGCCATGATT	TTTCGCTCTGGTCCGTCTTG	In line with that of the target gene	[241]
